# Neuroprotective Effect of Paeonol Mediates Anti-Inflammation via Suppressing Toll-Like Receptor 2 and Toll-Like Receptor 4 Signaling Pathways in Cerebral Ischemia-Reperfusion Injured Rats

**DOI:** 10.1155/2016/3704647

**Published:** 2016-12-22

**Authors:** Wen-Yen Liao, Tung-Hu Tsai, Tin-Yun Ho, Yi-Wen Lin, Chin-Yi Cheng, Ching-Liang Hsieh

**Affiliations:** ^1^Graduate Institute of Chinese Medicine, College of Chinese Medicine, China Medical University, Taichung 40402, Taiwan; ^2^Institute of Traditional Medicine, School of Medicine, National Yang-Ming University, Taipei 11221, Taiwan; ^3^School of Post-Baccalaureate Chinese Medicine, College of Chinese Medicine, Taichung 40402, Taiwan; ^4^School of Chinese Medicine, College of Chinese Medicine, China Medical University, Taichung 40402, Taiwan; ^5^Graduate Institute of Acupuncture Science, College of Chinese Medicine, China Medical University, Taichung 40402, Taiwan; ^6^Graduate Institute of Integrated Medicine, College of Chinese Medicine, China Medical University, Taichung 40402, Taiwan; ^7^Department of Chinese Medicine, China Medical University Hospital, Taichung 40447, Taiwan; ^8^Research Center for Chinese Medicine and Acupuncture, China Medical University, Taichung 40402, Taiwan

## Abstract

Paeonol is a phenolic compound derived from* Paeonia suffruticosa* Andrews (MC) and* P. lactiflora* Pall (PL). Paeonol can reduce cerebral infarction volume and improve neurological deficits through antioxidative and anti-inflammatory effects. However, the anti-inflammatory pathway of paeonol remains unclear. This study investigated the relationship between anti-inflammatory responses of paeonol and signaling pathways of TLR2 and TLR4 in cerebral infarct. We established the cerebral ischemia-reperfusion model in Sprague Dawley rats by occluding right middle cerebral artery for 60 min, followed by reperfusion for 24 h. The neurological deficit score was examined, and the brains of the rats were removed for cerebral infarction volume and immunohistochemistry (IHC) analysis. The infarction volume and neurological deficits were lower in the paeonol group (pretreatment with paeonol; 20 mg/kg i.p.) than in the control group (without paeonol treatment). The IHC analysis revealed that the number of TLR2-, TLR4-, Iba1-, NF-*κ*B- (P50-), and IL-1*β*-immunoreactive cells and TUNEL-positive cells was significantly lower in the paeonol group; however, the number of TNF-*α*-immunoreactive cells did not differ between the paeonol and control groups. The paeonol reveals some neuroprotective effects in the model of ischemia, which could be due to the reduction of many proinflammatory receptors/mediators, although the mechanisms are not clear.

## 1. Introduction

Stroke generally causes a permanent disability and is the second leading cause of death worldwide. Moreover, approximately 70% of patients experience ischemic stroke. The only approved medicine for ischemic stroke is recombinant tissue plasminogen activator (rt-PA) [1, 2]. However, the time window of rt-PA is narrow; thus, it should be injected within 3 h after stroke onset. Moreover, it can be administered only to a limited proportion of patients with stroke who meet strict criteria, and it increases the risk of intracranial hemorrhage in patients after receiving thrombolysis [3]. Increasing evidence has shown that thrombolysis alone is not adequate for ischemic stroke. The reperfusion phase may also create a second damage through aggravating inflammatory responses, oxidative stress, and brain edema [1, 4, 5]. Thus, a novel therapy that can improve the second damage progression should be developed.

Inflammation is an essential step in the progression of neural death after ischemic stroke, which is characterized by the production of cytokines, chemokines, and adhesion molecules that amplify tissue damage [6, 7]. Emerging in vivo and in vitro studies have highlighted that toll-like receptor (TLR) 2 (TLR2) and TLR4 have crucial roles in modulating postischemic inflammatory responses [8–10]. The extracellular domains of TLR2 and TLR4 can recognize both exogenous pathogen-associated molecular patterns (PAMPs) and endogenous damage-associated molecular patterns (DAMPs). Upon activation, microglial cells start expressing TLR2 and TLR4 on their surfaces. The binding of DAMPs to TLR2 and TLR4 initiates the signal transduction through myeloid differentiation factor 88 (MyD88). This leads to the degradation of inhibitor of kappa B (I*κ*B) and the triggering translocation of nuclear factor-*κ*B (NF-*κ*B) into the nucleus, which induces the production of proinflammatory cytokines such as interleukin-1*β* (IL-1*β*), IL-6, and tumor necrosis factor-*α* (TNF-*α*) [11]. Clinical studies have reported that the expression levels of TLR2 and TLR4 in peripheral blood in the acute stage are related to the severity and prognosis of patients in the following stage [12, 13].

Paeonol is a type of a phenolic compound isolated from* Paeonia suffruticosa* Andrews and* P. lactiflora* Pall. Paeonol possesses diverse biological activities including antioxidative, anti-inflammatory, and anticoagulative effects [14–16]. We previously reported that paeonol pretreatment can reduce infarction volume and improve neurological deficits by scavenging superoxide anions and inhibiting microglial activation and IL-1*β* production in cerebral ischemia-reperfusion injured rats [17]. However, no study has evaluated the effect of paeonol on TLR2 and TLR4 signaling pathways. Therefore, this study investigated whether paeonol can ameliorate inflammatory responses following cerebral ischemia-reperfusion injuries through the suppression of TLR2 and TLR4 signaling pathways. This study may increase our understanding about the neuroprotective mechanism of paeonol in cerebral ischemia-reperfusion injuries, which could be used to develop a novel agent for patients with stroke.

## 2. Materials and Methods

### 2.1. Animals

Adult male Sprague Dawley (SD) rats, weighing 290–320 g, were obtained from BioLASCO Co., Ltd., Taiwan. The rats were housed in the animal center of China Medical University (CMU) in a 12/12 h light-dark cycle at a constant temperature of 22 ± 2°C and humidity of 55%  ± 5%. They were provided ad libitum access to water and food. All experiments were performed following guidelines approved by the Institutional Animal Care and Use Committee of CMU.

### 2.2. Paeonol Preparation

**Figure 1:**
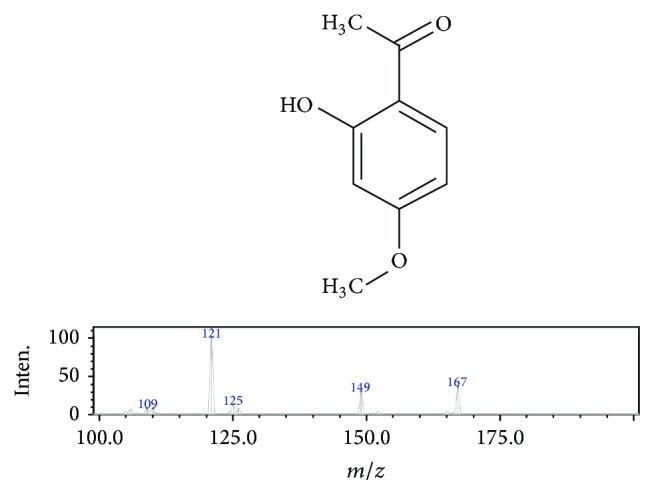
Spectral analysis of paeonol structure. Chemical names: 2′-hydroxy-4′-methoxyacetophenone; molecular formula: C_9_H_10_O_3_; molecular weight: 166.1739 g/mol; ionization mode: ESI (+); collision energy: −25.

Paeonol was extracted from the root bark of* P. suffruticosa* and the structure of paeonol was confirmed by NMR spectral analysis (Figure 1) in the laboratory room of Professor Tsai, Tung-Hu, National Yang-Ming University, Taipei, Taiwan. In summary, the root bark of* P. suffruticosa* was extracted with 95% ethanol, and then the extracts were combined and concentrated in vacuo, followed by partitioning against n-hexane that was described by Hsieh et al. (2006) [17]. The dose response study for this paeonol was reported in our previous study. Therefore, this study used this effective dose only [17]. The 10 mg of paeonol was dissolved in 150 *µ*L of glycofurol and then diluted in 4.85 mL of phosphate-buffered saline (PBS) to reach a final concentration of 2 mg/mL.

### 2.3. Establishment of Cerebral Ischemia-Reperfusion Injured Model

The intraluminal transient middle cerebral artery (MCA) occlusion model was established in SD rats as previously described [18]. Briefly, all animals were anesthetized using 5% isoflurane (Aerrane, Canada) and maintained using 2% isoflurane. A PE-50 catheter was introduced into the right femoral artery to monitor physiological parameters. After the midline neck incision, the right common carotid artery (CCA), ipsilateral internal carotid artery (ICA), and external carotid artery (ECA) were isolated from around the tissue, and the pterygopalatine artery was ligated close to its origin. A 3–0 nylon with a tip, blunted by a flame and coated with poly-L-lysine (UNIK, Taiwan), was inserted from the right ECA through the CCA and advanced into the ICA at a distance of 18–22 mm to block the right MCA origin. The right parietal bone was drilled to obtain a shallow hole, located 5 mm laterally and 1 mm posteriorly to the bregma, and the probe of Laser-Doppler flowmetry (DRT4, Moor Instruments Inc., Wilmington, USA) was placed inside the hole. When the cerebral blood flow of the right MCA distribution area decreased to less than 10% of the baseline value, the blood flow of the right MCA was blocked. The blood flow of the right MCA was blocked for 60 min, and then the nylon was removed to allow reperfusion.

### 2.4. Monitoring of Physiological Parameters

Rectal temperature, heart rate, arterial blood pressure, arterial blood gas (including pH, pO_2_, and pCO_2_), and blood sugar levels were measured 10 min before and 60 min after occluding the blood flow of the right MCA and 10 min after the reperfusion.

### 2.5. Grouping and Experiments

A total of 36 SD rats were randomly divided into the following three groups evenly: (1) sham group: the right CCA and ICA were exposed without occluding the blood flow of the right MCA; (2) control group: the methods were identical to those of the sham group; however, the blood flow of the right MCA was occluded for 60 min and then reperfusion was performed for 24 h; and (3) paeonol group: the methods were identical to those of the control group; however, paeonol (20 mg/kg) was intraperitoneally administrated 20 min prior to occluding the blood flow of the right MCA. Besides, both sham group and control group received administration with the same volume (10 mL/kg) of 3% glycofurol in PBS 20 min before the procedure.

All of 36 SD rats were evaluated for their neurological status 24 h after reperfusion, and then the rats were sacrificed to obtain their brains. Each group has 12 SD rats. The 6 SD rats of each group were used to measure their cerebral infarction volume, and the other 6 SD rats of each group were used for immunohistochemistry (IHC) and TUNEL assay study. Moreover, two rats of the control group were randomly selected for double immunofluorescence analysis.

### 2.6. Evaluation of Neurological Status

All of the 36 SD rats' neurological status were examined by a well-trained person who was blinded to the groups 24 h after the reperfusion according to the modified neurological severity score as described by Chen et al. [19]. The examination included motor test [raising the rat by the tail (0–3); placing the rat on the floor (0–3)], sensory test (0–2), beam balance test (0–6), and reflex test (0–4). The overall neurological function of each rat was graded from 0 to 18.

### 2.7. Measurement of Infarction Volume

After examining the neurological status, 6 SD rats of each group were anesthetized using chloral hydrate (400 mg/mL), followed by transcardial perfusion by using 200 mL of 0.9% saline. Then, the brains were removed, coronally sliced into 2 mm thick slices, stained using 2% 2,3,5-triphenyl tetrazolium chloride (TTC, Merck, Germany) for 15 min at 37°C, and fixed in 4% paraformaldehyde (PFA). Under the TTC staining, the infarcted brain tissue remains originally pale, whereas the viable tissue becomes deep red. The infarction and total brain volume were quantified using a microscopic image analysis software (ImageJ 1.47v, USA). The cerebral infarction volume was represented as a percentage of the infarcted tissue relative to the total brain tissue.

### 2.8. Immunohistochemistry

After examining the neurological status, 6 SD rats of each group were anesthetized using chloral hydrate (400 mg/mL) and transcardially perfused using 200 mL of 0.9% saline, followed by 200 mL of 4% PFA (pH 7.4). The brain tissues were postfixed in 4% PFA at pH 7.4 overnight and were transferred for dehydration in 30% sucrose for 3 days. The brains were embedded in a tissue-freezing medium (OCT compound, Triangle Biomedical Science, USA), frozen at −20°C, cut into 14-*μ*m coronal sections by using a cryostat (CM3050S, Leica, USA), and stored at −80°C. Six rats of each group were employed, and 20 slides of each rat were captured for IHC.

The brain sections were rinsed with PBS for 3 min and immersed in 3% H_2_O_2_/methanol for 15 min to inhibit endogenous peroxide activity. Thereafter, the sections were incubated with 10% normal animal serum (A kit, LsAB kit, Zymed, USA) for 10 min at room temperature. The sections were, respectively, incubated with primary anti-TLR2 (1 : 100 dilution, OriGene-Antibody, USA) overnight at 4°C, anti-TLR4 (1 : 200 dilution, Abcam, USA) overnight at 4°C, anti-Iba1 (1 : 1000 dilution, Abcam, USA) for 1 h at room temperature, anti-NF-*κ*B (p50; 1 : 200 dilution, Santa Cruz, USA) overnight at 4°C, anti-IL-1*β* (1 : 200 dilution, Santa Cruz, USA) overnight at 4°C, and anti-TNF-*α* (1 : 200 dilution, Santa Cruz, USA) overnight at 4°C and washed with PBS three times. After incubation with secondary antibodies (B kit, LsABkit, Zymed, USA) and the avidin-biotin peroxidase complex (C kit, LsAB kit, Zymed, USA), the sections were stained using 3,3-diaminobenzidine kit (Scytek Laboratories, USA) and counterstained with hematoxylin. The stained sections were mounted using mounting media (Assistant-Histokitt, Germany), and the number of immunoreactive cells, within the penumbra areas of 1 mm^2^, was calculated under a quick scan of ScanScope (CS2, Aperio, USA). The negative control stain was subjected to the same IHC assay on the adjacent section of the control group without active TLR2, TLR4, NF-*κ*B, IL-1*β*, and TNF-*α* antibody.

### 2.9. TUNEL Assay

Six SD rats of each group were used to TUNEL assay, which was used to detect DNA fragmentation in the penumbra areas. TUNEL staining was performed according to the manufacturer's instructions (Calbiochem). Briefly, the brain sections were incubated with 20 *μ*g/mL proteinase K for 20 min at room temperature, rinsed with TBS, incubated with 1x TdT equilibration buffer for 30 min at room temperature, and then incubated with TdT-labeling reaction mixture for 1.5 h at 37°C. After adding the stop solution and blocking buffer, the sections were incubated with 1x conjugate solution for 30 min at room temperature, and TUNEL-positive cells were visualized using the DAB kit. Finally, the sections were counterstained with methyl green and mounted using mounting media. The TUNEL-positive cells were calculated using the same method as that used in the IHC assay.

### 2.10. Double Immunofluorescence Analysis

Two SD rats of the control group were randomly selected for double immunofluorescence analysis. The brain sections were blocked for 10 min in PBS containing 10% bovine serum albumin (Sigma, USA). The sections were then incubated overnight at 4°C with primary antibodies [1 : 100 rabbit polyclonal anti-TLR2 (OriGene-Antibody, USA), 1 : 100 mouse monoclonal anti-TLR4 (Abcam, USA), and 1 : 500 goat polyclonal anti-Iba1 (Abcam, USA)]. Further, the sections were incubated with secondary antibodies [1 : 800 Alexa Fluor 488-conjugated donkey anti-rabbit, 1 : 800 Alexa Fluor 488-conjugated donkey anti-mouse, and 1 : 800 Cy™ 3-conjugated donkey anti-goat (Jackson ImmunoResearch Lab. Inc., USA)] for 1 h at room temperature. Each of the abovementioned steps was followed by three 3-min rinses in 0.01% Tween 20/PBS. At the end of the procedure, the sections were covered with coverslip using a mounting medium (SIGMA, USA) containing DAPI to counterstain DNA in the nuclei and dried overnight. The confocal images were captured using a laser-scanning confocal microscope (Leica TCS SP2, Germany).

### 2.11. Statistical Analysis

Data are expressed as mean ± standard deviation. Data from all the experimental groups were compared using one-way ANOVA followed by post hoc Scheffe's test. A probability value of less than 0.05 was considered statistically significant.

## 3. Results

### 3.1. Physiological Parameters

The physiological parameters including rectal temperature, heart rate, arterial blood pressure, arterial blood gas (pH, pO_2_, and pCO_2_), and blood sugar levels did not significantly differ among sham, control, and paeonol groups at 10 before and 60 min after occluding the blood flow of the right MCA and at 10 min after the reperfusion (all *p* > 0.05, Table 1).

### 3.2. Effect of Paeonol on Cerebral Infarction Volume and Neurological Deficits in Cerebral Ischemia-Reperfusion Injured Rats

**Figure 2:**
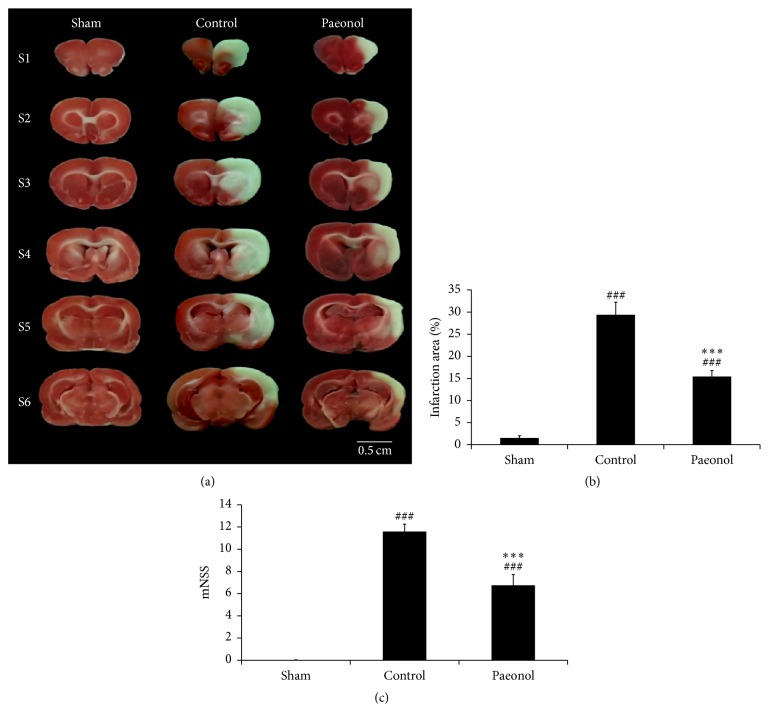
Effect of paeonol on cerebral infarction volume and neurological deficits in cerebral ischemia-reperfusion injured rats. (a) Representative photo showed TTC-stained brain slices in each group (*n* = 6). The infarcted tissue remained originally pale, whereas the viable tissue became deep red. (b) Quantitative analysis of cerebral infarction volume percentage (*n* = 6) showed smaller percentage in paeonol group compared with control group. (c) Quantitative analysis of modified neurological severity score (*n* = 12) in paeonol group was significantly lower than control group. Data are expressed as mean ± SD. ^###^*p* < 0.001 compared with the sham group; ^*∗∗∗*^*p* < 0.001 compared with the control group. Scale bar = 0.5 cm.

The control and paeonol groups exhibited varying cerebral infarction volume percentages after undergoing occlusion of the right MCA for 60 min, followed by reperfusion for 24 h (Figure 2(a)). The cerebral infarction volume percentages in the sham, control, and paeonol groups were 1.53% ± 0.48%, 29.39% ± 2.85%, and 15.42% ± 1.35%, respectively. The cerebral infarction volume percentages were higher in the control and paeonol groups than in the sham group (both *p* < 0.001, Figure 2(b)). Furthermore, compared with the control, treatment with paeonol significantly reduced the cerebral infarction volume percentage (*p* < 0.001; Figure 2(b)).

The rats in the control and paeonol groups developed different severities of neurological deficits after undergoing occlusion of the right MCA for 60 min, followed by reperfusion for 24 h. The modified neurological severity scores in the sham, control, and paeonol groups were 0.00 ± 0.00, 10.58 ± 0.67, and 6.57 ± 0.97, respectively. The modified neurological severity scores were higher in the control and paeonol groups than in the sham group (both *p* < 0.001, Figure 2(c)). The scores in the paeonol group were significantly lower than those in the control group (*p* < 0.001; Figure 2(c)).

### 3.3. Effect of Paeonol on the Number of TLR2-, TLR4-, and Iba1-Immunoreactive Cells within the Cortical Penumbra in Cerebral Ischemia-Reperfusion Injured Rats

**Figure 3:**
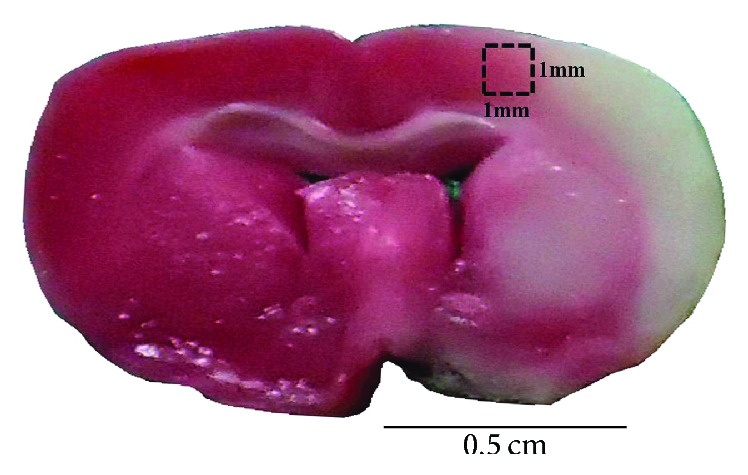
Representative photo showed the brain coronal section located 0.72 mm posterior to the bregma position. The dashed line square, located 2 mm laterally to the midline, within the cerebral penumbral zone indicated the region for calculating immunoreactive cells. The length and width of dashed line square = 1 mm. Scale bar = 0.5 cm.

All immunoreactive cells were counted within the dotted line area of 1 mm^2^ (counts/1 mm^2^; Figure 3).

**Figure 4:**
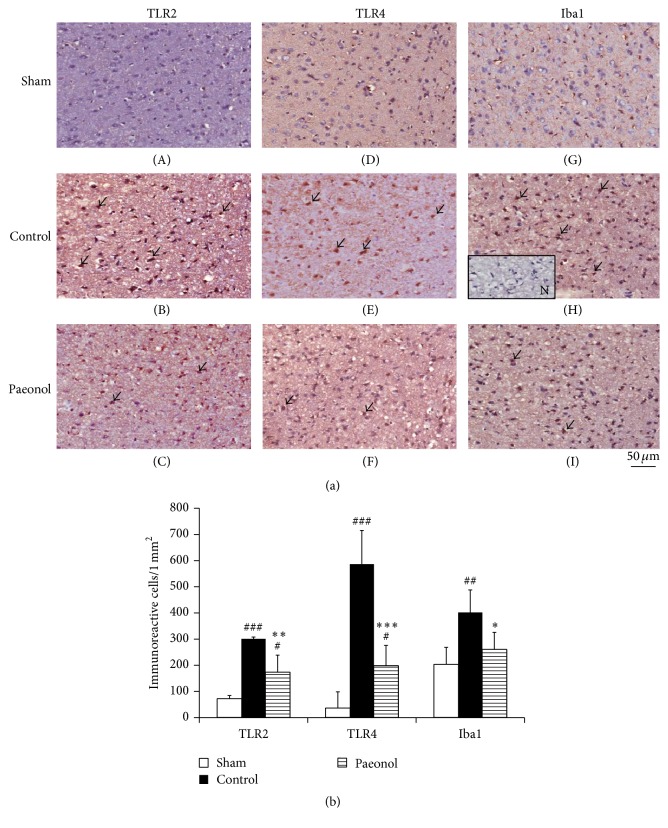
Effect of paeonol on the number of TLR2-, TLR4-, and Iba1-immunoreactive cells within the cortical penumbra in cerebral ischemia-reperfusion injured rats. (a) The numbers of TLR2-, TLR4-, and Iba1-immunoreactive cells (arrows) were lower in the paeonol group than in the control group. (200x); (b) the numbers of TLR2-, TLR4-, and Iba1-immunoreactive cells were significantly lower in the paeonol group than in the control group (*n* = 6). Data are expressed as mean ± SD. ^###^*p* < 0.001, ^##^*p* < 0.01, and ^#^*p* < 0.05 compared with the sham group; ^*∗∗∗*^*p* < 0.001, ^*∗∗*^*p* < 0.01, and ^*∗*^*p* < 0.05 compared with the control group. N: negative control; sham: sham group; control: control group; paeonol: paeonol group.

The number of TLR2-immunoreactive cells in the sham, control, and paeonol groups was 70.8 ± 13.2, 301.2 ± 63.3, and 172.5 ± 65.9, respectively. The number of TLR2-immunoreactive cells was significantly higher in the control and paeonol groups than in the sham group (*p* < 0.001 and *p* < 0.05, respectively; Figures 4(a) and 4(b)). Moreover, there were significantly fewer immunoreactive cells in the paeonol group than in the control group (*p* < 0.01; Figures 4(a) and 4(b)).

The number of TLR4-immunoreactive cells in the sham, control, and paeonol groups was 35.2 ± 7.0, 586.7 ± 128.7, and 197.3 ± 86.3, respectively. The number of TLR4-immunoreactive cells was significantly higher in the control and paeonol groups than in the sham group (*p* < 0.001 and *p* < 0.05, respectively; Figures 4(a) and 4(b)). Moreover, there were significantly fewer immunoreactive cells in the paeonol group than in the control group (*p* < 0.001; Figures 4(a) and 4(b)).

The number of Iba1-immunoreactive cells in the sham, control, and paeonol groups was 202.3 ± 66.4, 402.0 ± 78.5, and 259.5 ± 66.0, respectively. The number of Iba1-immunoreactive cells was significantly lower in the paeonol group than in the control group (*p* < 0.05; Figures 4(a) and 4(b)), and it did not significantly differ between the sham and paeonol groups (*p* > 0.05; Figures 4(a) and 4(b)).

### 3.4. Effect of Paeonol on the Number of NF-*κ*B-, IL-1*β*-, and TNF-*α*-Immunoreactive Cells within the Cortical Penumbra in Cerebral Ischemia-Reperfusion Injured Rats

**Figure 5:**
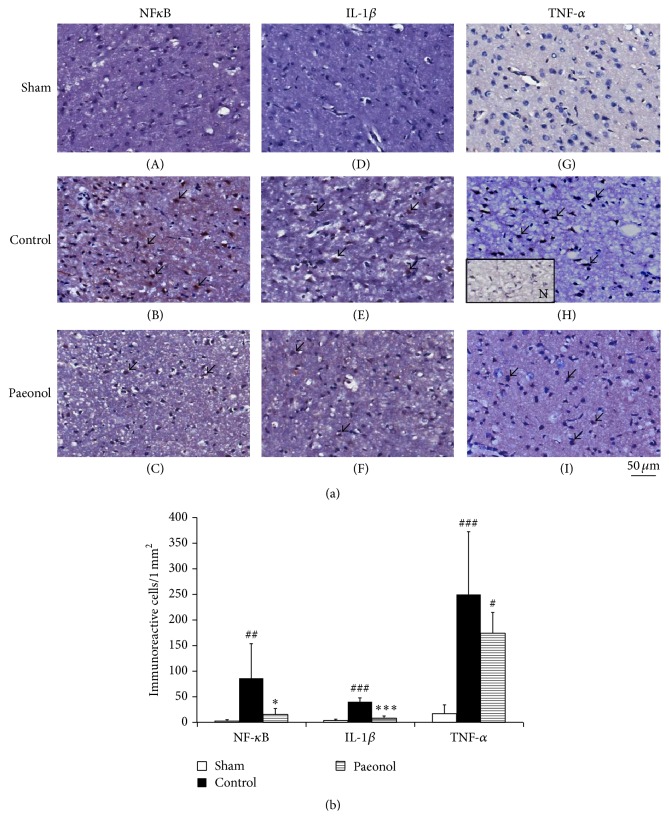
Effect of paeonol on the number of NF-*κ*B-, IL-1*β*-, and TNF-*α*-immunoreactive cells within the cortical penumbra in cerebral ischemia-reperfusion injured rats. (a) The numbers of NF-*κ*B- and IL-1*β*-immunoreactive cells (arrows) were lower in the paeonol group than in the control group, whereas the number of TNF-*α*-immunoreactive cells (arrows) did not differ between the paeonol and control groups (200x); (b) the numbers of NF-*κ*B- and IL-1*β*-immunoreactive cells were significantly lower in the paeonol group than in the control group (*n* = 6), whereas it did not significantly differ between the paeonol and control groups. Data are expressed as mean ± SD. ^###^*p* < 0.001 and ^##^*p* < 0.01, and ^#^*p* < 0.05 compared with the sham group; ^*∗∗∗*^*p* < 0.001 and ^*∗*^*p* < 0.05 compared with the control group. N: negative control; sham: sham group; control: control group; paeonol: paeonol group.

The number of NF-*κ*B-immunoreactive cells in the sham, control, and paeonol groups was 2.7 ± 2.6, 86.0 ± 67.7, and 15.2 ± 11.7, respectively. The number of NF-*κ*B-immunoreactive cells was significantly lower in the paeonol group than in the control group (*p* < 0.05; Figures 5(a) and 5(b)), and it did not significantly differ between the sham and paeonol groups (*p* > 0.05; Figures 5(a) and 5(b)).

The number of IL-1*β*-immunoreactive cells in the sham, control, and paeonol groups was 3.7 ± 2.7, 40.2 ± 7.8, and 7.8 ± 4.5, respectively. The number of IL-1*β*-immunoreactive cells was significantly lower in the paeonol group than in the control group (*p* < 0.001; Figures 5(a) and 5(b)), and it did not significantly differ between the sham and paeonol groups (*p* > 0.05; Figures 5(a) and 5(b)).

The number of TNF-*α*-immunoreactive cells in the sham, control, and paeonol groups was 17.0 ± 20.7, 249.7 ± 122.8, and 173.8 ± 40.7, respectively. The number of TNF-*α*-immunoreactive cells was significantly higher in the control and paeonol groups than in the sham group (*p* < 0.001 and *p* < 0.05, respectively; Figures 5(a) and 5(b)), and it did not significantly differ between the control and paeonol groups (*p* > 0.05; Figures 5(a) and 5(b)).

### 3.5. Effect of Paeonol on TUNEL-Positive Cells within the Cortical Penumbra in Cerebral Ischemia-Reperfusion Injured Rats

**Figure 6:**
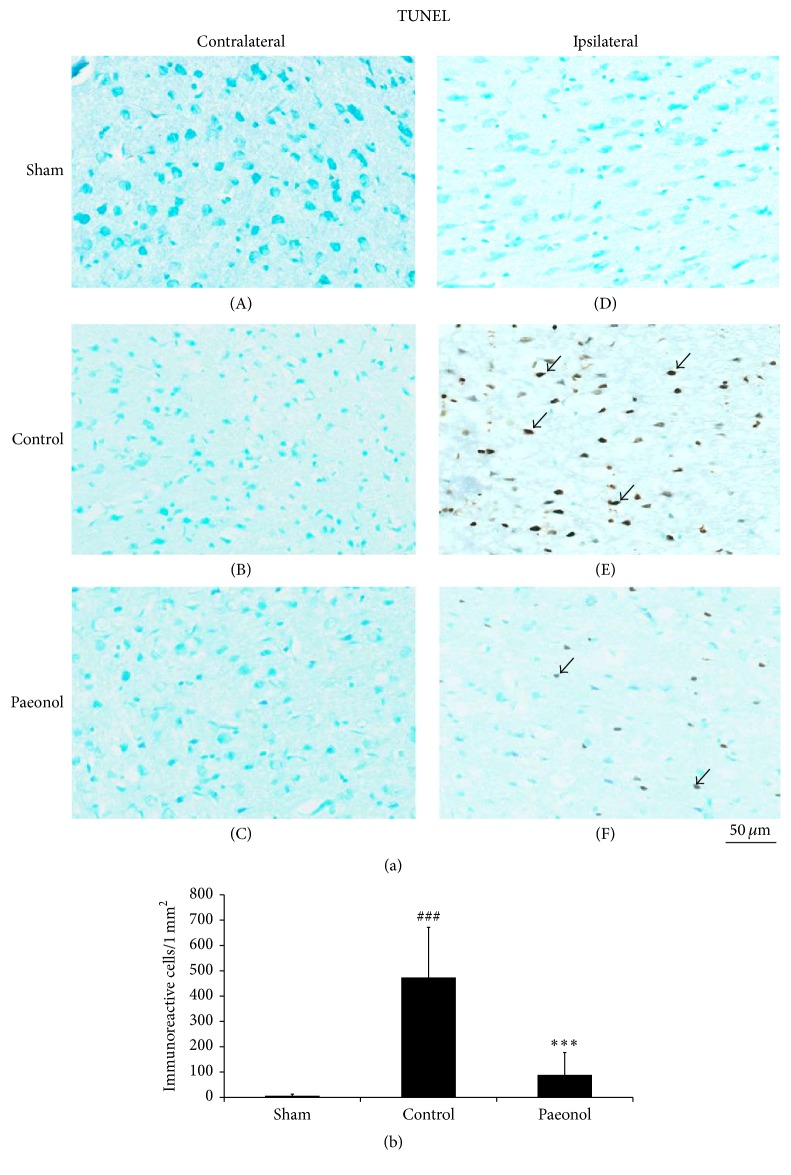
Effect of paeonol on apoptosis within the cortical penumbra in cerebral ischemia-reperfusion injured rats. (a) No TUNEL-positive cells were observed in the sham group and contralateral hemisphere. The number of TUNEL-positive cells (arrows) was lower in the paeonol group than in the control group. (b) The number of TUNEL-positive cells was significantly lower in the paeonol group than in the control group (*n* = 6). Data are expressed as mean ± SD. ^###^*p* < 0.001 compared with the sham group; ^*∗∗∗*^*p* < 0.001 compared with the control group. Sham: sham group; control: control group; paeonol: paeonol group.

TUNEL assay was used to detect apoptotic cells 24 h after reperfusion. The number of TUNEL-positive cells in the sham, control, and paeonol groups was 6.5 ± 6.2, 473.7 ± 198.2, and 88.8 ± 97.7, respectively, in the ipsilateral hemisphere. No TUNEL-positive cells were observed in the contralateral hemisphere (Figures 6(a) and 6(b)). The number of TUNEL-positive cells was significantly lower in the paeonol group than in the control group in the ipsilateral hemisphere (*p* < 0.001; Figures 6(a) and 6(b)), and it did not significantly differ between the sham and paeonol groups in the ipsilateral hemisphere (*p* > 0.05; Figures 6(a) and 6(b)).

### 3.6. Effect of Paeonol on Double Immunofluorescence Analysis in Cerebral Ischemia-Reperfusion Injured Rats

**Figure 7:**
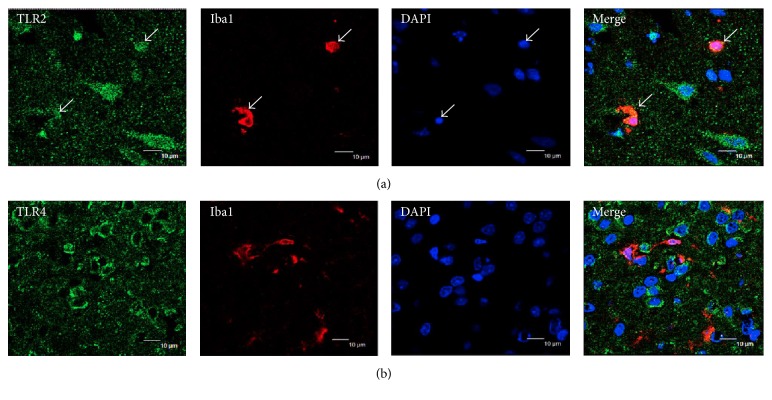
Effect of paeonol on double immunofluorescence analysis in cerebral ischemia-reperfusion injured rats. (a) Double immunofluorescence analysis revealed the colocalization of TLR2-immunoreactive cells (green) with the microglia marker Iba1 (red, colocalization marked with white arrows); (b) no colocalization of TLR4 (green) with Iba1 (red) was observed (*n* = 2). Scale bar = 10 *μ*m.

To evaluate whether TLR2 and TLR4 are related to activating microglia after the ischemia-reperfusion injury, double immunofluorescence analysis was performed. The double immunofluorescence analysis of the brain sections, adjacent to the sections used for IHC analysis, revealed the colocalization of TLR2 staining with Iba1 in the cortical penumbra 24 h after reperfusion (Figure 7(a)). By contrast, no colocalization of TLR4 staining with Iba1 was observed (Figure 7(b)).

## 4. Discussion

The results of the present study indicated that paeonol pretreatment can reduce cerebral infarction volume; neurological deficits; TLR2-, TLR4-, Iba1-, NF-*κ*B-, and IL-1*β*-immunoreactive cell numbers; and TUNEL-positive cells in cerebral ischemia-reperfusion injured rats. These results are consistent with those of our previous study that paeonol pretreatment can reduce cerebral infarction volume and neurological deficits, scavenge superoxide anions, and inhibit microglial activation and IL-1*β* production [17]. Inflammation is an essential step in the progression of neural damage after cerebral ischemia-reperfusion injuries, which is characterized by the production of cytokines, chemokines, and adhesion molecules that amplify tissue damage [20]. Emerging clinical and experimental studies have highlighted the key roles of TLR2 and TLR4 in microglia, which contribute to inflammatory responses occurring after cerebral ischemia-reperfusion injuries [21–23]. The expression of TLR2 and TLR4 in microglia contributes to neuronal damage in cerebral ischemia-reperfusion injuries [21–23]. Moreover, accumulating evidence indicated that the effective suppression of TLR2 and TLR4 signaling pathways in microglia can provide neuroprotection [24–28].

Microglia are resident macrophage-like cells in the central nervous system [29]. During an ischemic stroke, microglia can be activated within minutes and reaches a peak after 2 to 3 days [30]. Microglia has both detrimental and beneficial effects at different time points. In early activation, microglia produces inflammatory substances leading to cell death, whereas in later activation, it produces transforming growth factor-beta 1 and acts as a neuroprotective agent [22, 30]. Upon activation, microglia begins expressing TLR2 and TLR4 on its surface. In response to DAMPs, which are released by necrotic cells, the signal transduction of TLR2 and TLR4 is induced through MyD88, resulting in the degradation of I*κ*B and the translocation of NF-*κ*B into the nucleus; this induces the production of proinflammatory cytokines such as IL-1*β*, IL-6, and TNF-*α* [11, 20]. Several studies have reported the effective inhibition of TLR2 and TLR4 expression in different models that exhibited decreased NF-*κ*B activity and proinflammatory cytokine suppression corresponding with decreased tissue damage [24, 25, 27, 28].

IL-1*β* can be observed 2–6 h after cerebral ischemia and reaches a peak at 12–24 h [31, 32]. IL-1*β* is considered to be a neurotoxic mediator, which induces neuronal apoptosis and enhances chemokine expression in microglia and astrocytes [4, 31, 33]. Moreover, the inhibition of IL-1*β* production reduces infarction volume [32, 34]. In the present study, a significantly higher number of Iba1-, TLR2-, TLR4-, NF-*κ*B-, IL-1*β*-, and TNF-*α*-immunoreactive cells and TUNEL-positive cells were observed in the control group, which is in accordance with the results of previous studies. In addition, paeonol pretreatment significantly reduced the number of Iba1-, TLR2-, TLR4-, NF-*κ*B-, and IL-1*β*-immunoreactive cells and TUNEL-positive cells. Therefore, these results suggest that paeonol pretreatment can attenuate inflammatory responses through the suppression of TLR2 and TLR4 signaling pathways to ameliorate apoptosis within the penumbral area.

In our study, TNF-*α* levels did not significantly differ between the paeonol and control groups. TNF-*α* is another proinflammatory cytokine involved in cerebral ischemia-reperfusion injuries. TNF-*α* gradually increases from 1 to 3 h after cerebral ischemia and reaches a peak at 24–36 h [35–37]. Several studies have reported that TNF-*α* can function both as a neurotoxic and as a neuroprotective mediator [38, 39]. TNF-*α* executes its neurotoxic function by promoting the production of oxygen free radicals and excitatory amino acids, which ultimately results in neuronal cell death. TNF-*α* deficiency and anti-TNF-*α* antibodies exert neuroprotective effects [39, 40]. By contrast, another study reported that TNF-*α*-knockout mice have a higher infarction volume [41]. It appears that TNF-*α* promotes inflammatory responses and is involved in the suppression of inflammatory signal transduction; in other words, TNF-*α* may regulate both proinflammation and anti-inflammation after cerebral ischemia-reperfusion injuries [41]. In the present study, a higher number of TNF-*α*-immunoreactive cells and lower infarction volume and neurological deficits were observed in the paeonol group. However, the present study was limited only to 24 h after reperfusion. Therefore, a long-term observational study is required to understand the role of TNF-*α* in ischemic stroke treated with paeonol.

TLR2 is mainly upregulated in lesion-associated microglia 24–72 h after cerebral ischemia [42] but is also observed in astrocytes and endothelial cells [28]. Similar to TLR2, TLR4 is also significantly upregulated in microglia and astrocytes 24 h after cerebral ischemia [43]. Moreover, neurons express TLR2 and TLR4 1 h after cerebral ischemia-reperfusion injuries [23, 42]. In our study, we observed the colocalization of TLR2 staining with Iba1 in the cortical penumbra area in the control group, which is in agreement with the results of the abovementioned studies. By contrast, no colocalization of TLR4 staining with Iba1 was observed in the control group. Our findings indicated that the inflammatory responses of microglia within the cortical penumbra may mainly occur through the TLR2 signaling pathway, whereas those in other brain cells, such as astrocytes and neurons, or in other ischemic sites, such as the cortical ischemic core or striatum, may occur through the TLR4 signaling pathway. However, additional studies are required in the future to validate these findings.

Some limitations in the present study because the study used immunochemistry images alone are sometimes not enough to provide quantitative measurements. We will perform to complement these data with immunoblotting or ELISA, in particular, TLR2, TLR4, and NF-*κ*B expression levels in the future.

In conclusion, paeonol pretreatment ameliorated cerebral infarction and neurological deficits, probably by reducing many proinflammatory receptors and mediators in cerebral ischemia-reperfusion injured rats, although the mechanisms are not clear.

## Figures and Tables

**Table 1:** Physiological parameters.

	BT (°C)	HR (beats/min)	MABP (mmHg)	pH	pO_2_ (mmHg)	pCO_2_ (mmHg)	BS (mg/dl)
*Preischemia 10 min*							
Sham	36.9 ± 0.5	328.3 ± 27.1	96.9 ± 12	7.41 ± 0.04	401.0 ± 94.6	41.3 ± 8.3	128.6 ± 26.6
Control	37.2 ± 0.5	357.0 ± 46.2	100.8 ± 8.8	7.43 ± 0.03	411.9 ± 54.7	42.6 ± 4.5	139.4 ± 34.3
Paeonol	36.9 ± 0.5	346.8 ± 51.1	97.4 ± 9.5	7.42 ± 0.03	434.3 ± 49.6	41.9 ± 4.8	131.9 ± 30.3
*Ischemia 60 min*							
Sham	34.8 ± 0.7	323.6 ± 69.3	90.3 ± 10.7	7.29 ± 0.12	394.1 ± 97.2	56.3 ± 18.9	115.1 ± 29.4
Control	35.2 ± 0.6	364.3 ± 46.0	92.9 ± 10.0	7.32 ± 0.07	408.9 ± 51.9	52.5 ± 12.0	123.4 ± 36.4
Paeonol	35.1 ± 0.7	346.3 ± 44.9	90.2 ± 7.90	7.33 ± 0.07	404.3 ± 62.6	49.0 ± 11.0	120.1 ± 28.6
*Reperfusion 10 min*							
Sham	35.3 ± 0.9	317.9 ± 65.2	96.4 ± 19.7	7.33 ± 0.10	378.7 ± 128.7	53.0 ± 16.1	53.0 ± 16.1
Control	35.5 ± 0.8	346.3 ± 55.4	93.1 ± 9.3	7.34 ± 0.06	412.3 ± 73.2	50.9 ± 9.6	50.9 ± 9.6
Paeonol	35.5 ± 0.5	348.9 ± 33.0	95.3 ± 5.9	7.34 ± 0.06	360.8 ± 110.3	43.4 ± 9.1	43.4 ± 9.1

Physiological parameters are expressed as mean ± SD (*n* = 12 for each group) and were measured 10 min before and 60 min after occluding the blood flow of the right middle cerebral artery and 10 min after reperfusion. Sham: sham group; control: control group; paeonol: paeonol group; BT: body temperature; HR: heart rate; MABP: mean arterial blood pressure; BS: blood sugar.
